# Synthesis and Photovoltaics of Novel 2,3,4,5-Tetrathienylthiophene-co-poly(3-hexylthiophene-2,5-diyl) Donor Polymer for Organic Solar Cell

**DOI:** 10.3390/polym13010002

**Published:** 2020-12-22

**Authors:** Morongwa E. Ramoroka, Siyabonga B. Mdluli, Vivian S. John-Denk, Kwena D. Modibane, Christopher J. Arendse, Emmanuel I. Iwuoha

**Affiliations:** 1 SensorLab (University of the Western Cape Sensor Laboratories), Chemical Sciences Building, Robert Sobukwe Road, Bellville, Cape Town 7535, South Africa; 3693152@myuwc.ac.za (M.E.R.); 3680647@myuwc.ac.za (S.B.M.); 2 Department of Chemistry, School of Physical and Mineral Science, University of Limpopo, Sovenga, Polokwane 0727, South Africa; kwena.modibane@ul.ac.za; 3 Department of Physics and Astronomy, University of the Western Cape, Bellville, Cape Town 7535, South Africa; cjarendse@uwc.ac.za

**Keywords:** donor polymer, organic solar cells, poly(3-hexylthiophene-2,5-diyl), 2,3,4,5-tetrathienylthiophene-co-poly(3-hexylthiophene-2,5-diyl)

## Abstract

This report focuses on the synthesis of novel 2,3,4,5-tetrathienylthiophene-co-poly(3-hexylthiophene-2,5-diyl) (TTT-co-P3HT) as a donor material for organic solar cells (OSCs). The properties of the synthesized TTT-co-P3HT were compared with those of poly(3-hexylthiophene-2,5-diyl (P3HT). The structure of TTT-co-P3HT was studied using nuclear magnetic resonance spectroscopy (NMR) and Fourier-transform infrared spectroscopy (FTIR). It was seen that TTT-co-P3HT possessed a broader electrochemical and optical band-gap as compared to P3HT. Cyclic voltammetry (CV) was used to determine lowest unoccupied molecular orbital (LUMO) and highest occupied molecular orbital (HOMO) energy gaps of TTT-co-P3HT and P3HT were found to be 2.19 and 1.97 eV, respectively. Photoluminescence revealed that TTT-co-P3HT:PC_71_BM have insufficient electron/hole separation and charge transfer when compared to P3HT:PC_71_BM. All devices were fabricated outside a glovebox. Power conversion efficiency (PCE) of 1.15% was obtained for P3HT:PC_71_BM device and 0.14% was obtained for TTT-co-P3HT:PC_71_BM device. Further studies were done on fabricated OSCs during this work using electrochemical methods. The studies revealed that the presence of poly(3,4-ethylenedioxythiophene) polystyrene sulfonate (PEDOT:PSS) on the surface of indium tin oxide (ITO) causes a reduction in cyclic voltammogram oxidation/reduction peak current and increases the charge transfer resistance in comparison with a bare ITO. We also examined the ITO/PEDOT:PSS electrode coated with TTT-co-P3HT:PC_71_BM, TTT-co-P3HT:PC_71_BM/ZnO, P3HT:PC_71_BM and P3HT:PC_71_BM/ZnO. The study revealed that PEDOT:PSS does not completely block electrons from active layer to reach the ITO electrode.

## 1. Introduction

Organic-based solar cells (OSCs) have attained considerable attention in the last decades and have been greatly studied because of their advantageous properties such as light weight, inexpensive, attractive colors and flexibility [1,2,3,4]. In comparison with traditional inorganic-based solar cell devices, OSCs have attracted research interest due to their constantly increasing power conversion efficiency (PCE) and solution processability [5]. The continuous improvement of OSCs is because of better understanding of the light conversion mechanism, development of novel materials and architectures of these devices [6,7,8]. To date, the OSCs with better performance are constructed on bulk heterojunction active layer using conjugated polymers as the electron donor materials and fullerenes as the electron acceptor materials [6,9,10]. The donor and acceptor material interface delivers a pathway for photo-generated charges, transportation and separation. Currently, the research in the OSCs field focuses on designing low energy band gap donor polymers with engineered electrochemical energy levels for better charge separation which lead to an increase in the performance of OSCs [11,12,13].

Organic donor materials play a crucial part on the overall performance of OSCs, and can be easily modified chemically. Polythiophenes based polymers are mostly used as the electron donor materials for OSCs because of their properties such as thermally stable, environmentally friendly, easy to process and less expensive [14,15]. These polythiophenes based polymers are widely reported [14,15,16,17]. Katsumata et al. [16] reported OSCs with maximum PCE of 2.03% using poly[2,6-(4,4-bis-(2-ethylhexyl)-4H-cyclopenta[2,1-b;3,4-b’]dithiophene)-alt-4,7(2,1,3-benzothiadiazole)] (PCPDTBT) as organic donor material. Kesters et al. [17] chemically modified PCPDTBT with 4,4-dialkyl-4H-cyclopenta[2,1-b;3,4-b′]dithiophene derivative with ester and alcohol moieties to achieve PCE of 2.02% and 1.82% respectively. Whereas the unmodified PCPDTBT possessed PCE of 2.15% [17]. The studies above showed that modification of organic donor materials has an effect on the PCE of OSCs.

Poly(3-hexylthiophene-2,5-diyl (P3HT) is the most widely studied polythiophene-based material as organic donor for solar cells [18,19,20,21,22] Studies revealed that P3HT properties such as optoelectronic properties and crystallinity and its OSCs performance can be altered by modification of its end groups [18,19,20]. Seibers et al. [21] reported P3HT functionalized with porphyrin as an end group for use as OSC donor material. Their findings revealed that porphyrin-functionalized P3HT have similar crystallinity, improved absorption properties and better OSCs performance when compared to pristine P3HT. Lim et al. [22] investigated morphological changes and OSCs performance of their synthesized hydrophobic end-functionalized P3HT. Repulsive interactions between hydrophobic end-functionalized P3HT and hydrophilic PCBM provided large continuous interfacial area. As a result, improvement in OSCs performance was achieved because of sufficient electron/hole dissociation. Hydrophobic end-functionalized P3HT maintained the crystallinity of P3HT. Bromo end-functionalized P3HT has a wider optical band gap, different crystallinity and lower OSCs performance as compared to P3HT. Bromo bonded to thiophene acted as exciton quenching and charge trapping sites leading to lower OSCs performance [23]. Properties of P3HT are very sensitive to the presence of the end groups.

In this work, we synthesized a novel TTT-co-P3HT donor material using oxidation polymerization. The performance of TTT-co-P3HT was compared with that of a commercial P3HT. From the conventional device fabricated during this study, the performance of P3HT based device was better than that of TTT-co-P3HT based device due to insufficient electron/hole separation and charge transfer of TTT-co-P3HT material. In both OSCs, PEDOT:PSS was used as an interlayer between ITO and active layer. The OSCs fabricated during this work were further investigated using electrochemical methods.

## 2. Experimental Section

### 2.1. Materials

Chemicals and solvents used in this study were procured from commercial sources and used without any further purification. Silica gel 60 Å (230–400 mesh, Merck (Pty) Ltd., Johannesburg, South Africa) was used for purification.

### 2.2. Synthesis of 2,3,4,5-Tetrathienylthiophene (TTT)

In a 100 mL round bottom flask, 2,3,4,5-tetrabromothiophene (1.00 g, 2.5 mmol, 99.0%, Merck (Pty) Ltd.), 2-thienylboronoic acid (1.28 g, 10 mmol, ≥95.0%, Merck (Pty) Ltd.) and potassium carbonate (K_2_CO_3_, 2.76 g, 20 mmol, 99.5%, Merck (Pty) Ltd.) were stirred for 30 min in 40 mL degassed tetrahydrofuran (THF, 99.0%, Merck (Pty) Ltd.):water solution with a volume ratio of 4:1. In the solution above, palladium tetrakis(triphenylphosphine) (Pd(PPh_3_)_4_, 0.29 g, 0.20 mmol, 99.0%, Merck (Pty) Ltd.) was added. The mixture was refluxed under nitrogen atmosphere for 24 h at 110 °C before being cooled to room temperature. Afterwards, the resulting solution was poured in a separating funnel containing 50 mL of brine, followed by extraction 3 times with dichloromethane (DCM, 99.9%, Merck (Pty) Ltd.) and 3 times with diethyl ether (≥99.0%, Merck (Pty) Ltd.). The extracts were dried with magnesium sulphate (≥99.5%, Merck (Pty) Ltd.), filtered and rotary evaporator (Buchi AG, Flawil, Switzerland) was used to remove the solvent. Column chromatography was used to purify the residue with DCM and methanol (MeOH, 99.5%, Merck (Pty) Ltd.) as an eluent (DCM:MeOH = 3:1, *v/v*) resulting to a yellow solid (0.76 g) with a yield of 74%. ^1^H-NMR (CDCl_3_, Merck (Pty) Ltd., 400 MHz): δ_H_ 6.89–6.91 (dd, 2H), 6.93–6.96 (m, 4H), 7.09–7.10 (dd, 2H), 7.19–7.21 (dd, 2H), 7.29–7.31 (dd, 2H); ^13^C-NMR (CDCl_3_, 400 MHz): δ_C_ 126.36, 126.42, 126.84, 126.95, 127.15, 129.40, 132.42, 133.33, 135.37, 135.67.

### 2.3. Synthesis of TTT-co-P3HT

In a typical chemical polymerization, TTT (0.12 g, 0.28 mmol) and 3-hexylthiophene (1.87 g, 11.12 mmol, ≥99.0%, Merck (Pty) Ltd.) were added in 50 mL of chloroform (≥99.0%, Merck (Pty) Ltd.). The solution was stirred for 30 min and followed by addition of iron chloride (FeCl_3_, 1.8 g, 11.12 mmol, ≥99.99%, Merck (Pty) Ltd.). The mixture was refluxed at 60 °C for 24 h under nitrogen atmosphere, then polymerization was terminated by addition of MeOH. After, the precipitates were collected by filtration and were washed with Soxhlet extraction using acetone (99.3%, Merck (Pty) Ltd.) and MeOH as solvents for 24 h with each. Finally, the product was extracted with DCM for another 24 h. The DCM solvent was removed to obtain a dark brown solid (0.36 g).

### 2.4. Characterization Techniques

The UV-Vis spectra (Nicolet Evolution 100 from Thermo Electron Corporation, Altrincham, UK), photoluminescence spectra (NanoLog with FluorEssence™ V3 software from Horiba Jobin Yvon, Edison, NJ, USA), size exclusion chromatogram (Agilent 1260 Infinity series HPLC instrument from Agilent Technologies, Waldbronn, Germany), nuclear magnetic resonance spectra (Bruker 400 MHz Avance III HD Nanobay spectrometer, Karlsruhe, Germany) and attenuated total reflectance-FTIR spectra (Spectrum-100 FTIR spectrometer from PerkinElmer (Pty) Ltd., Midrand, South Africa) were recorded with the specified instruments. Device characteristics (current density–voltage) were measured with Keithley 2420 Source Meter (Keithley Instruments B.V., Gorinchem, Netherlands) using an illumination of AM 1.5G, 100 mW cm^−2^ supplied by a solar simulator (Sciencetech Inc., London, ON, Canada).

CV and electrochemical impedance spectroscopy (EIS) of the thin films were measured in 0.1 M tetrabutylammonium hexafluorophosphate (TBAPF_6_, ≥99.0%, Merck (Pty) Ltd.) in acetonitrile (99.8%, Merck (Pty) Ltd.) as supporting electrolyte. Three electrodes cell system consist of ITO (15–25 Ω/square resistance, Merck (Pty) Ltd.), platinum wire (Goodfellow Cambridge Ltd., Huntingdon, UK) and silver/silver chloride (Ag/AgCl, from BASi^®^ in West Lafayette, IN, USA) as working electrode, counter electrode and reference electrode respectively were used. Ferrocene (98%, Merck (Pty) Ltd.) was used as a redox probe molecule. All the electrochemical measurements were conducted using a Bio-Logic VMP-300 (Bio-Logic SAS, Seyssinet-Pariset, France). The zview software (Scribner Associates Inc., Southern Pines, NC, USA) was used to fit EIS results.

### 2.5. Fabrication of OSCs

Devices fabrication and measurements were conducted in air. ITO (8 pixels, 20 Ω/square resistance, Ossila Ltd., Sheffield, UK) substrates were sonicated in 1% (by volume) hellmanex III (Merck (Pty) Ltd.) solution in hot water, followed by in acetone then 2-propylalcohol for 5 min with each. After every sonication step, the substrate was rinsed with water. PEDOT:PSS (0.5–1.0 wt % in water, Merck (Pty) Ltd.) was sonicated for 3 h then filtered with 0.45 μm filter. It (30 μL PEDOT:PSS) was spun coated on ITO substrate for 30 s at 4000 rpm. The film was baked at 150 °C for 5 min. Active layer was prepared by blending donor material polymer and PC_71_BM (mixture of isomers, 99%, Merck (Pty) Ltd.) with a total of 25 mg/mL in chlorobenzene (99.9%, Merck (Pty) Ltd.) as a solvent at 60 °C for overnight. The blend ratio of donor: acceptor used is 1:0.8. The solutions of 40 μL were spun at 2000 rpm for 30 s in air. The films were annealed for 5 min at 100 °C. ZnO solution (preparation method is given in supporting document) of 30 μL was spun coated at 4000 rpm for 30 s and was annealed at 100 °C for 30 min. Lastly, aluminum metal (Merck (Pty) Ltd.) was thermally evaporated at deposition pressure of about 10^−5^ mbar to complete the device.

These fabrication conditions were used to prepare thin films for characterization. The deposition of TTT-co-P3HT, TTT-co-P3HT:PCBM (1:0.8), P3HT (99.995%, Merck (Pty) Ltd.) and P3HT:PCBM (1:0.8) on ITO substrates with/without PEDOT:PSS were done outside glovebox.

## 3. Results and Discussion

**Scheme 1:**
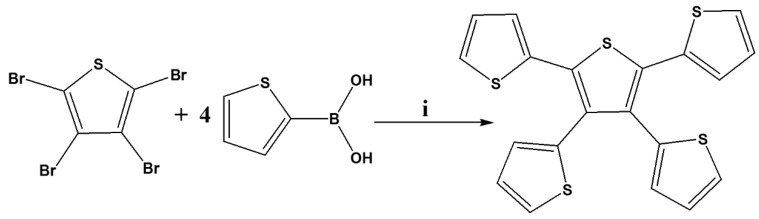
Synthetic route of 2,3,4,5-tetrathienylthiophene. (i) 10% Pd(PPh_3_)_4_, K_2_CO_3_, THF/water (4:1), 110 °C, 24 h.

**Scheme 2:**
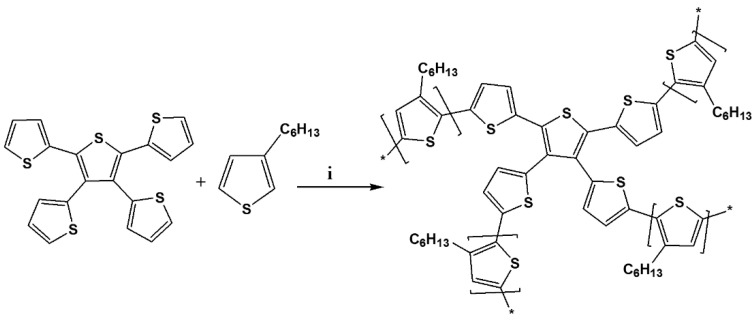
Synthesis of TTT-co-P3HT. (i) FeCl_3_, 60 °C, 24 h.

**Figure 1:**
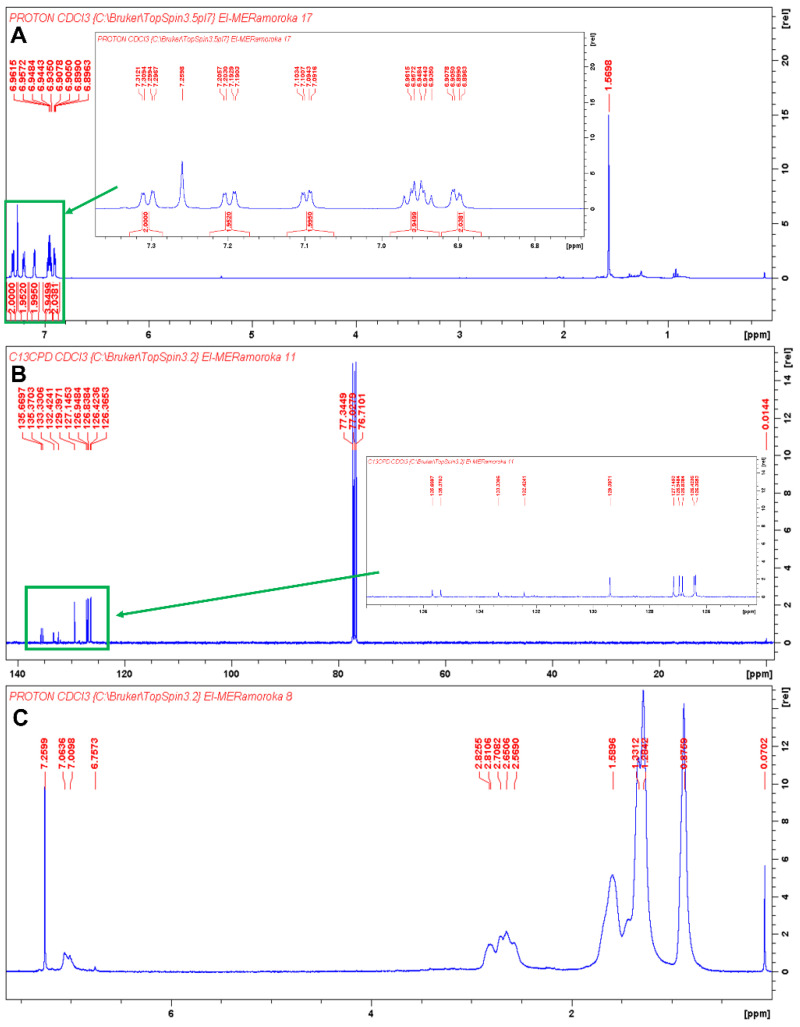

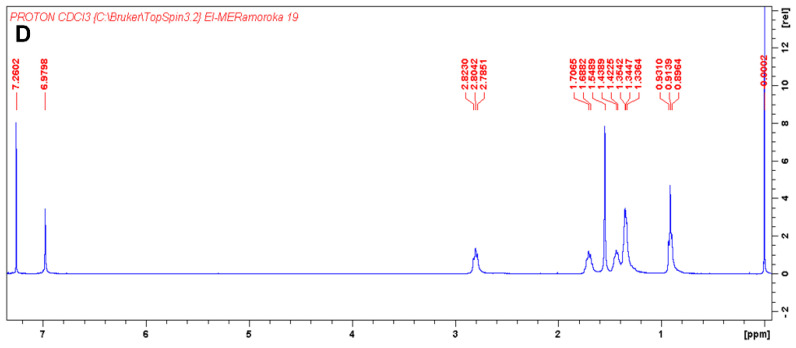
(**A**) ^1^H-NMR of TTT in CDCl_3_ and the insertion is ^1^H-NMR of TTT in the chemical shift range of 6.74 to 7.38 ppm. The signal at 1.5698 ppm is due to the present of water; (**B**) ^13^C-NMR of TTT in CDCl_3_ and the insertion is ^13^C-NMR of TTT in the chemical shift range of 127 to 138 ppm; (**C**) ^1^H-NMR of TTT-co-P3HT in CDCl_3_ and (**D**) ^1^H-NMR of P3HT CDCl_3_.

As shown in Scheme 1 and Scheme 2, synthesis of TTT and TTT-co-P3HT were performed by using Suzuki coupling and oxidation polymerization reaction methods, respectively. Results of size exclusion chromatography are shown in Table 1 and the chromatogram of TTT-co-P3HT is shown in Figure S1. The synthesized polymer, TT-co-P3HT, has a molecular weight Mn of 15,131 gmol^−1^ and a polydispersity index of 3.6. The chemical structure of TTT and TTT-co-P3HT were confirmed by NMR. The ^1^H-NMR of TTT in Figure 1A reveals no presence of –OH protons from 2-thienylboronoic acid confirming successful synthesis. The spectrum was designed at a chemical shift range between 6.74 and 7.38 ppm as an insertion in Figure 1A. Number of protons obtained by integrating the signals corresponds to the number of protons from the chemical structure of TTT. Figure 1B shows the ^13^C-NMR spectra of TTT and due to structural symmetry, only 10 signals were observed. For TTT-co-P3HT, only ^1^H-NMR analysis was performed (Figure 1C) and compared with the ^1^H-NMR spectrum of P3HT (Figure 1D). The ^1^H-NMR spectrum of TTT-co-P3HT showed a new signal at 6.75 ppm which is absent on the ^1^H-NMR spectrum of P3HT. This signal is due to the present of β-hydrogens from TTT. The signals from ^1^H-NMR spectrum of TTT-co-P3HT are broader than those of P3HT. This is due to different P3HT chain lengths attached to TTT.

**Figure 2:**
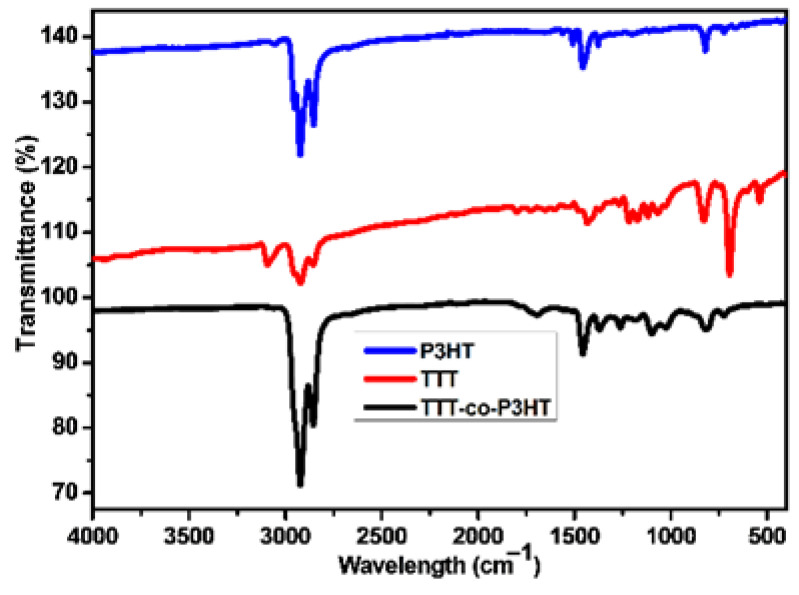
FTIR of P3HT, TTT-co-P3HT, and TTT.

FTIR spectra of TTT, TTT-co-P3HT and P3HT were recorded from 4000 cm^−1^ to 400 cm^−1^. The spectra are shown in Figure 2. Unique vibrational bands can be observed at the wavenumber of 696 cm^−1^, the range from 2934 to 2844 cm^−1^ and 3085 cm^−1^ on the spectrum of TTT. The vibrational band at 696 cm^−1^ is due to the aromatic C–H out of phase bending vibrations of thiophene [24,25,26], while the vibrational bands in the range from 2934 to 2844 cm^−1^ are due to the β-position C–H of the thiophene. The presence of the α-position C–H is confirmed by the vibrational band at the wavenumber of 3085 cm^−1^ [26,27]. In comparison with the spectra of P3HT and TTT-co-P3HT, the vibrational bands in the range from 2934 to 2844 cm^−1^ increases in intensity due to the presence of the hexyl group. The vibrational bands at 696 cm^−1^ and 3085 cm^−1^ disappears due to the occurrence of polymerization.

### 3.1. Optical and Electrochemical Characterization

**Figure 3:**
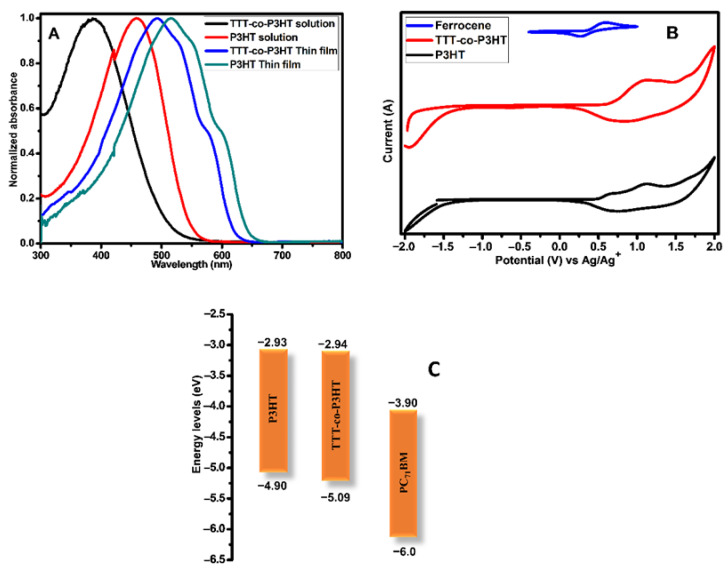
(**A**) Absorption spectra; (**B**) cyclic voltammetry (CV) and (**C**) energy levels diagram of P3HT and TTT-co-P3HT.

UV-Vis spectra of TTT-co-P3HT and P3HT materials are presented in Figure 3A and were obtained in chlorobenzene solvent and as thin films. The data obtained from Figure 3A is given in Table 2. As thin films, TTT-co-P3HT and P3HT materials have maximum absorption at a longer wavelength as compared to the spectra obtained in chlorobenzene. This is an indication that intermolecular π–π stacking is stronger in thin films. In both chlorobenzene and thin films, TTT-co-P3HT showed maximum absorbance at shorter wavelengths. This shows that TTT disturbs the interchain delocalization of π-electrons of P3HT after functionalization [23]. The onset absorption wavelengths were used to determine optical band-gaps of TTT-co-P3HT and P3HT. The optical band gaps for TTT-co-P3HT were determined to be 2.32 eV and 1.98 eV in chlorobenzene and thin film, respectively. As for P3HT, they were determined to be 2.21 eV in chlorobenzene and 1.91 eV in thin film. TTT-co-P3HT have broader band gaps indicating that lower number of photons are absorbed. Therefore, decreased numbers of electron/hole pairs are generated and TTT-co-P3HT OSCs is expected to have a decreased short circuit current-density (Jsc).

CV was used to investigate electrochemical response of TTT-co-P3HT and P3HT donor polymers and the voltammograms are shown in Figure 3B. Table 2 displays the results of HOMO and LUMO energy levels obtained from Figure 3B. These energy levels offsets of the donor and acceptor materials are important factors in understanding the electron/hole pair separation dynamics. HOMO offset becomes of importance when the acceptor material absorbs light significantly [28,29]. In this work, we focus only on the LUMO offset. The LUMO offsets between donor and acceptor are determined from Figure 3C to be 0.96 eV for TTT-co-P3HT:PC_71_BM and 0.97 eV for P3HT:PC_71_BM. The values obtained for LUMO offsets are more than 3 times higher than the commonly known empirical threshold of 0.3 eV [30]. Gadisa et al. [31] studied the relationship between onset oxidation potentials of polythiophene derivatives and open circuit voltage (Voc). Their studies revealed that Voc decreases as onset oxidation potential increases. Gao et al. [32] reported donor and acceptor materials with LUMO offset less than threshold. They achieved low energy loss and high Voc. Therefore, TTT-co-P3HT:PC_71_BM and P3HT:PC_71_BM have high energy loss and this will have an impact on the inVoc.

**Figure 4:**
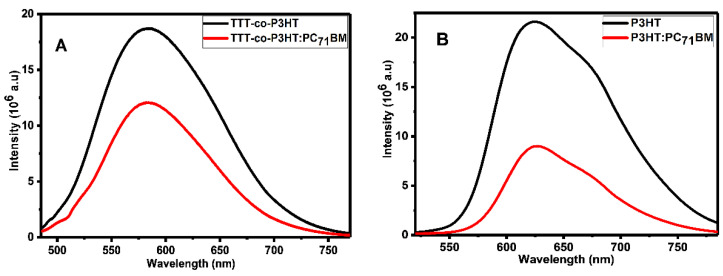
Photoluminescence spectra of (**A**) TTT-co-P3HT and TTT-co-P3HT:PC_71_BM and (**B**) P3HT and P3HT:PC_71_BM.

Photoluminescence spectroscopy has been widely used to study electron/hole pair separation at the interface of donor and acceptor materials using the quenching effect [33,34,35,36]. Figure 4 depicts the photoluminescence results of (A) TTT-co-P3HT and TTT-co-P3HT:PC_71_BM and (B) P3HT and P3HT:PC_71_BM obtained using chlorobenzene as a solvent. Photoluminescence quenching was observed in both TTT-co-P3HT:PC71BM and P3HT:PC71BM blends. This quenching can be attributed to charge transfer and electron/hole pair separation efficiency which corroborates other results in literature [34]. To determine the quenching degree of TTT-co-P3HT:PC_71_BM and TTT-co-P3HT:PC_71_BM, the following Equation (1):

(1) $$q\left(\%\right)=\frac{I_{\mathrm{donor}}-I_{\mathrm{donor}:\mathrm{acceptor}}}{I_{\mathrm{donor}}}\times 100$$

was used to calculate photoluminescence quenching parameter q, where I_donor_ is the intensity of donor material and I_donor:acceptor_ is the intensity of donor:acceptor blend [33]. From Equation (1), for an outstanding degree of electron/hole pair separation and charge transfer without any recombination taking place, I_donor:acceptor_ must be equal to zero resulting to q equal to 100%. John et al. [35] achieved q of 97.29% and 96.6% indicating excellent electron/hole separation and charge transfer for their blends. The quenching parameter q was found to be 36% in TTT-co-P3HT:PC_71_BM and 58% in P3HT:PC_71_BM. This reveals that some of the created electron/hole pair recombine in TTT-co-P3HT and P3HT [36]. When comparing two blends, the results show that electron/hole pair separation and charge transfer is sufficient in P3HT:PC_71_BM than in TTT-co-P3HT:PC_71_BM.

### 3.2. Photovoltaic Properties

**Figure 5:**
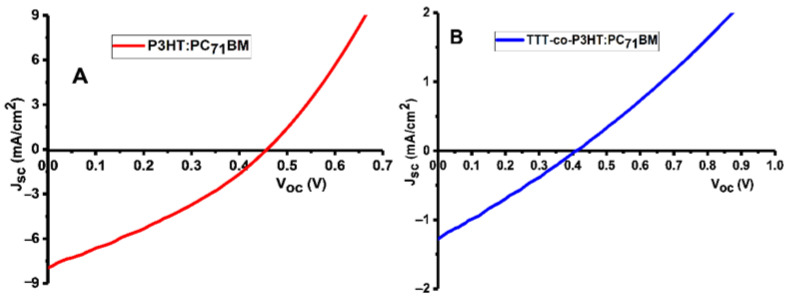
J–V plots of (**A**) P3HT:PC_71_BM and (**B**) TTT-co-P3HT:PC_71_BM devices.

OSCs were fabricated during this study with conventional configuration as follows: ITO/PEDOT:PSS/TTT-co-P3HT:PC_71_BM/ZnO/Al and ITO/PEDOT:PSS/P3HT:PC_71_BM/ZnO/Al. Figure 5 depicts the current-density (J) versus voltage (V) plots of (A) P3HT:PC_71_BM and (B) TTT-co-P3HT:PC_71_BM as active layers. The solar cells parameters obtained for the devices are recorded in Table 3. The organic bulk heterojunction solar cell containing TTT-co-P3HT produced smallest J_SC_ (1.27 mA/cm^2^), V_OC_ (0.41 V), fill factor, FF (26.78%) and PCE (0.14%) in comparison with the device containing P3HT which produced the largest J_SC_ (7.91 mA/cm^2^), V_OC_ (0.46 V), FF (31.64%) and PCE (1.15%). The improved performance of the device fabricated using P3HT was ascribed to sufficient electron/hole pair separation confirmed by photoluminescence and lower band-gap in comparison with TTT-co-P3HT. Low Jsc and Voc in TTT-co-P3HT based OSC is attributed to the disruption of the ordered lamellar stacking of P3HT by modification with TTT. This disruption results to a decrease in absorption and low hole mobility in TTT-co-P3HT [37,38]. P3HT and TTT-co-P3HT based OSCs were fabricated in air. Therefore, oxygen permeation does occur and will oxidize low work function aluminum electrode. Oxidized aluminum electrode will form a charge transport barrier. This induces S-shaped I–V curve and reduces the performance of the OSCs [39]. Additionally, penetrative oxygen in the active layer lead to different photo-oxidation reactions of an acceptor and donor materials [40,41]. Changes in the structures of an acceptor and donor materials will change their charge carrier mobilities, energy levels and photon absorption properties. The oxygen doping in the active layer will increase the concentration of holes, which results in an increase in trapping of electrons and a decrease in Voc and FF [42,43].

### 3.3. Characterization of OSCs with Electrochemical Methods

**Figure 6:**
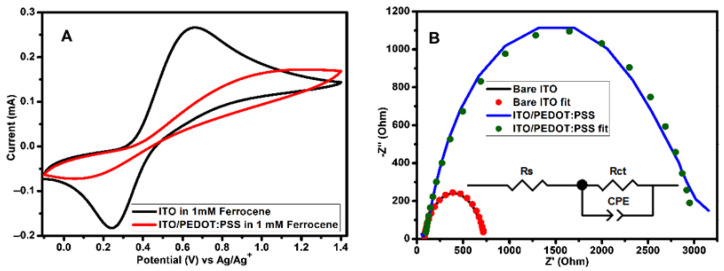
(**A**) CV (measured at 100 mVs^−1^ scan rate) and (**B**) Nyquist plots of 1 mM ferrocene obtained using bare ITO and ITO/(PEDOT:PSS as working electrodes in 0.1 M TBAPF_6_/acetonitrile solution. The Nyquist plots were attained at bias potential of 0.45 V vs Ag/Ag^+^. The equivalent circuit used for impedance data fitting is shown as an inset on (**B**). ITO—indium tin oxide; PEDOT:PSS—poly(3,4-ethylenedioxythiophene) polystyrene sulfonate.

In order to further investigate the OSCs fabricated during this work, electrochemical techniques methods were used. ITO coated substrate was used as working electrode during electrochemical studies. The layers on the ITO substrates were prepared using OSCs fabrication conditions. Electrochemical techniques such as CV and EIS can be used to quantitatively study the electron-blocking ability of PEDOT:PSS interlayer. Parameters such as peak separation and peak current are very useful in evaluating electron-blocking properties because these parameters depend strongly on the amount of electroactive species from the electrolyte that are exposed to electroactive sites of the working electrode. Figure 6 depicts CV and Nyquist plots of ITO without/with PEDOT:PSS layer in 1 mM ferrocene prepared using 0.1 M TBAPF_6_ in acetonitrile. CV (Figure 6A) obtained in the presence of PEDOT:PSS layer show a decrease in the oxidation/reduction peak currents and an increase in peak separations. The peak currents for a bare ITO is 0.27 mA for cathodic peak (I_pc_) and for anodic peak (I_pa_) is −0.18 mA, while for ITO/PEDOT:PSS are I_pc_ is 0.17 mA and I_pa_ is −0.07 mA. The extent to which the peak currents decreased after coating PEDOT:PSS onto the ITO substrate were estimated using Equation (2):

(2) $$I_{\mathrm{decreased}}=\frac{I_{\mathrm{without}}-I_{\mathrm{with}}}{I_{\mathrm{without}}}$$

where I_decreased_ is the amount of current decreased, I_without_ is the peak current of bare ITO and I_with_ is the peak current of ITO/PEDOT:PSS [44]. The values of I_decreased_ was found to be 0.54 for cathodic peaks and 0.82 for anodic peaks. The peak separation for bare ITO was determined to be 0.42 V and for ITO/PEDOT:PSS was determined to be 1.17 V. The decrease in peak currents and an increase in peak separation for ITO/PEDOT:PSS suggest a decrease in the ITO electrode activity. These observations indicate that the PEDOT:PSS interlayer does not completely block electroactive species from ferrocene to reach ITO surface since the value of I_decreased_ is not equal to 1. Therefore, PEDOT:PSS interlayer does not completely block electrons when is used as hole transport interlayer in OSCs and this might also be due to the presence of pinholes.

EIS is an important instrument that can be used to research charge transfer processes at the interlayer [45,46,47] and evaluate the surface coverage at the electrode active area [44,48]. Figure 6B shows the Nyquist plots of bare ITO and ITO/PEDOT:PSS with the equivalent circuit as an inset. In the circuit, R_s_ is an Ohmic resistance, R_ct_ is a resistance of charge transfer processes taking place at the interface and constant phase element (CPE) proposes a non-ideal behavior of the capacitor. CPE is well-defined by two adjustable values (CPE-T and CPE-P) and is mostly used as a capacitor-like element to compensate interfacial inhomogeneity (surface states or defects). In the case where CPE-P is equal to 1, then CPE and ideal capacitor are identical without defects [47,49,50]. The plots were fitted with zview software and the results of parameters obtained are recorded in Table 4. The value of R_ct_ for bare ITO was found to be 655.10 Ω and for ITO/PEDOT:PSS was found to be 2935.00 Ω. These results reveal that PEDOT:PSS block the diffusion of electroactive species from ferrocene containing supporting electrolyte to the ITO surface. The electrode surface coverage (θ) can be estimated from R_ct_ of bare ITO and ITO/PEDOT:PSS using Equation (3):

(3) $$\theta =1-\frac{R_{\mathrm{ct}}^{\mathrm{bare}\;\mathrm{ITO}}}{R_{\mathrm{ct}}^{\mathrm{ITO}/\mathrm{PEDOT}:\mathrm{PSS}}}$$

where R_ct_^bare ITO^ and R_ct_^ITO/PEDOT:PSS^ are charge transfer resistances measured at bare ITO and ITO/PEDOT:PSS, respectively [48]. If the surface is completely covered, the value R_ct_^ITO/PEDOT:PSS^ must be too big in such a way that θ will close to 1 [44]. From the R_ct_ values attained by fitting the Nyquist plots, a value of θ = 0.78 was determined. Therefore, this indicate the presence of pinholes.

**Figure 7:**
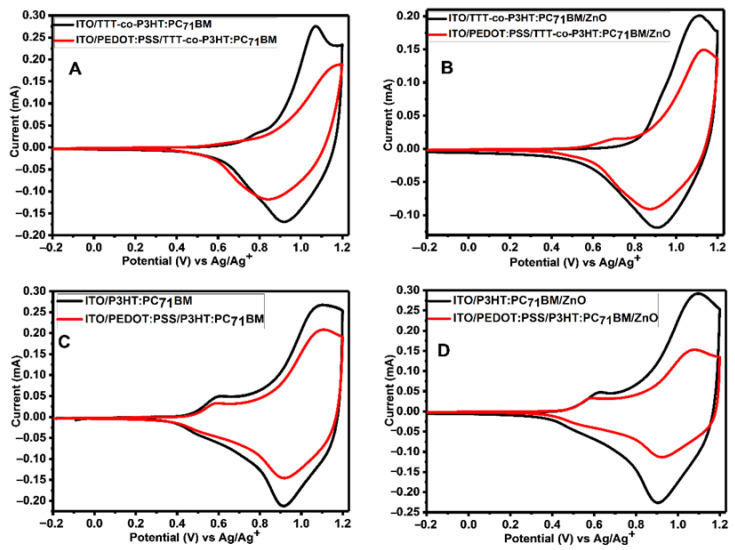
CV of (**A**) TTT-co-P3HT:PC_71_BM, (**B**) PEDOT:PSS/TTT-co-P3HT:PC_71_BM, (**C**) P3HT:PC_71_BM and (**D**) PEDOT:PSS/P3HT:PC_71_BM thin films coated on ITO substrate with/without PEDOT:PSS interlayer at 100 mVs^−1^ in 0.1 M TBAPF_6_/acetonitrile solution.

Figure 7 depicts CV of active layers (TTT-co-P3HT:PC_71_BM and P3HT:PC_71_BM) and active layers/ZnO coated onto a bare ITO and ITO/PEDOT:PSS electrodes. The results show that the presence of PEDOT:PSS cause a decrease in the peak currents. Cathodic and anodic peak currents are used to study the degree at which the peak current decreased using Equation (2). The results obtained are shown in Table 5. The extent at which the cathodic peak current is reduced because of PEDOT:PSS interlayer presence is 0.29 for TTT-co-P3HT:PC_71_BM, 0.25 for TTT-co-P3HT:PC_71_BM/ZnO, 0.29 for P3HT:PC_71_BM and 0.52 for P3HT:PC_71_BM/ZnO. While amount at which an anodic peak current decreased was determined to be 0.32 for TTT-co-P3HT:PC_71_BM, 0.25 for TTT-co-P3HT:PC_71_BM/ZnO, 0.22 for P3HT:PC_71_BM and 0.48 for P3HT:PC_71_BM/ZnO. According to Equation (2), if an interlayer does completely block electroactive species from reaching the surface of the electrode, the value of I_decreased_ must be equal to 1. These obtained values are low not only because of poor electron blocking properties of PEDOT:PSS, but due to the presence of pinholes on the PEDOT:PSS interlayer which allow active layer to be in direct contact with ITO electrode.

## 4. Conclusions

In summary, we have successfully synthesized TTT-co-P3HT using chemical oxidation polymerization for use as the donor material in OSCs. The properties of TTT-co-P3HT were compared with those of pristine P3HT. The optical band gaps of TTT-co-P3HT and P3HT in chlorobenzene were found to be 2.32 eV and 2.21 eV respectively. The LUMO offsets of active layers TTT-co-P3HT:PC_71_BM and P3HT:PC_71_BM were determined to be 0.96 eV and 0.97 eV, respectively. The TTT-co-P3HT:PC_71_BM active layer has insufficient electron/hole pair separation and charge transfer at the interface, which was confirmed by photoluminescence quenching studies. The OSCs device of P3HT:PC_71_BM exhibited a better performance with an efficiency of 1.15%, while TTT-co-P3HT:PC_71_BM exhibited an efficiency of 0.14%. Poor performance of TTT-co-P3HT in OSC is because of its low hole mobility and decreased photon absorbance due to the disturbance in ordered lamellar stacking of P3HT after functionalization. To further understand the deviation in TTT-co-P3HT:PC_71_BM and P3HT:PC_71_BM devices performance, we studied the layers of OSCs fabricated during this work using electrochemical methods. The study revealed that PEDOT:PSS interlayer does not completely block electrons from active layer to the ITO substrate. From cyclic voltammetry results, the diffusion of electrons to the ITO substrate was observed by a decrease in the current of the peaks. Therefore, this study gives an opportunity to further optimize OSCs using cheap and reliable electrochemical methods and also shows the importance of using electrochemical methods in study the interlayers behavior for OSCs use.

## Figures and Tables

**Table 1:** Size exclusion chromatography analysis of TTT-co-P3HT.

Polymer	Mn (g·mol^−1^)	Mw (g·mol^−1^)	Mp (g·mol^−1^)	Mz (g·mol^−1^)	Polydispersity Index	Mz/Mw
TTT-co-P3HT	15,131	54,421	38,613	157,867	3.60	2.90

**Table 2:** Optical and electrochemical responses of TTT-co-P3HT and P3HT.

Polymer	^a^ λ (nm)		^c^ E_g_^optical^ (eV)	^b^ λ (nm)		^c^ E_g_^optical^ (eV)	^d^ E_Homo_ (eV)	^d^ E_Lumo_ (eV)	^e^ E_g_^CV^ (eV)
	Max	Onset		Max	Onset				
TTT-co-P3HT	386	534	2.32	492	626	1.98	−5.09	−2.90	2.19
P3HT	458	561	2.21	516	650	1.91	−4.90	−2.93	1.97

^a^ In chlorobenzene solution. ^b^ In thin film. ^c^ E_g_^opt^ = 1240/λ_onset_ eV in the thin film. ^d^ Obtained with CV method, E_LUMO_ = −[(E_oxidation-onset_ − E_Ferrocene_) + 4.8] eV and E_HOMO_ = −[(E_reduction-onset_ − E_Ferrocene_) + 4.8]. ^e^ E_g_^CV^ was estimated using the equation: E_g_^CV^ = E_LUMO_ − E_HOMO_.

**Table 3:** Response of the TTT-co-P3HT:PC_71_BM and P3HT:PC_71_BM devices.

Active Layer	J_sc_ (mA/cm^2^)	V_oc_ (V)	FF (%)	PCE (%)
TTT-co-P3HT:PC_71_BM	1.27	0.41	26.78	0.14
P3HT:PC_71_BM	7.91	0.46	31.64	1.15

**Table 4:** Results obtained from fitting electrochemical impedance data of bare ITO and ITO/PEDOT:PSS.

	R_s_ (Ω)	R_ct_ (Ω)	CPE-T (μF)	CPE-P (n)
ITO	75.57 ± 0.06	655.10 ± 4.05	19.33 ± 0.51	0.82 ± 0.01
ITO/PEDOT:PSS	85.31 ± 2.15	2935.00 ± 39.65	18.53 ±0.68	0.82 ± 0.01

**Table 5:** Cathodic and anodic peak currents obtained from CV used to study the effect of PEDOT:PSS interlayer.

	^a^ I_pa (without)_ mA	^b^ I_pc (without)_ mA	^c^ I_pa (with)_ mA	^d^ I_pc (with)_ mA	^e^ I_decreased (pa)_	^f^ I_decreased (pc)_
TTT-co-P3HT:PC_71_BM	0.28	−0.17	0.19	−0.12	0.32	0.29
TTT-co-P3HT:PC_71_BM/ZnO	0.20	−0.12	0.15	−0.09	0.25	0.25
P3HT:PC_71_BM	0.27	−0.21	0.21	−0.15	0.22	0.29
P3HT:PC_71_BM/ZnO	0.29	−0.23	0.15	−0.11	0.48	0.52

^a^ I_pa (without)_ is an anodic peak current obtained without PEDOT:PSS interlayer, ^b^ I_pc (without)_ is cathodic peak current obtained without PEDOT:PSS interlayer, ^c^ I_pa (with)_ is an anodic peak current obtained with PEDOT:PSS interlayer, ^d^ I_pc (with)_ is cathodic peak current obtained with PEDOT:PSS interlayer, ^e^ I_decreased (pa)_ is the extent at which anodic peak current decreased and ^f^ I_decreased (pc)_ is an extent at which cathodic peak current decreased.

## Supplementary Materials

The following are available online at https://www.mdpi.com/2073-4360/13/1/2/s1, Figure S1: Size exclusion chromatography analysis of TTT-co-P3HT in THF.

## References

[1] Qing L., Zhong A., Chen W., Cao Y., Chen J. (2020). Largely improved bulk-heterojunction morphology in organic solar cells based on a conjugated terpolymer donor via a ternary strategy. Polymer.

[2] Genene Z., Negash A., Abdulahi B.A., Eachambadi R.T., Liu Z., Brande N.V.D., D’Haen J., Wang E., Vandewal K., Maes W. (2020). Comparative study on the effects of alkylsilyl and alkylthio side chains on the performance of fullerene and non-fullerene polymer solar cells. Org. Electron..

[3] Li X., Li K., Su D., Shen F., Huo S., Fu H., Zhan C. (2020). Design a thieno[3,2-b]thiophene bridged nonfullerene acceptor to increase open-circuit voltage, short-circuit current-density and fill factor via the ternary strategy. Chin. Chem. Lett..

[4] Huang K., Li M., He M., Liang Z., Geng Y. (2020). Difluorobenzoxadiazole-based conjugated polymers for efficient non-fullerene polymer solar cells with low voltage loss. Org. Electron..

[5] Li T., Chen Z., Wang Y., Tu J., Deng X., Li Q., Li Z. (2019). Materials for interfaces in organic solar cells and photodetectors. ACS Appl. Mater. Interfaces.

[6] Chen T.L., Zhang Y., Smith P., Tamayo A., Liu Y., Ma B. (2011). diketopyrrolopyrrole-containing oligothiophene-fullerene triads and their use in organic solar cells. ACS Appl. Mater. Interfaces.

[7] Niklas J., Zheng T., Nashchadin A., Mardis K.L., Yu L., Poluektov O.G. (2019). Polaron and exciton delocalization in oligomers of high-performance polymer PTB7. J. Am. Chem. Soc..

[8] Liang Y., Wu Y., Feng D., Tsai S.-T., Son H.-J., Li G., Yu L. (2009). Development of new semiconducting polymers for high performance solar cells. J. Am. Chem. Soc..

[9] Ranjitha A., Thambidurai M., Shini F., Muthukumarasamy N., Velauthapillai D. (2019). Effect of doped TiO_2_ film as electron transport layer for inverted organic solar cell. Mater. Sci. Energy Technol..

[10] Revoju S., Biswas S., Eliasson B., Sharma G.D. (2018). Effect of acceptor strength on optical, electrochemical and photovoltaic properties of phenothiazine-based small molecule for bulk heterojunction organic solar cells. Dye. Pigment..

[11] Cui Y., Yao H., Zhang J., Zhang T., Wang Y., Hong L., Xian K., Xu B., Zhang S., Peng J. (2019). Over 16% efficiency organic photovoltaic cells enabled by a chlorinated acceptor with increased open-circuit voltages. Nat. Commun..

[12] Zhao W., Li S., Yao H., Zhang S., Zhang Y., Yang B., Hou J. (2017). Molecular optimization enables over 13% efficiency in organic solar cells. J. Am. Chem. Soc..

[13] Li S., Ye L., Zhao W., Zhang S., Mukherjee S., Ade H., Hou J. (2016). Energy-level modulation of small-molecule electron acceptors to achieve over 12% efficiency in polymer solar cells. Adv. Mater..

[14] Ansari M.A., Mohiuddin S., Kandemirli F., Malik M.I. (2018). Synthesis and characterization of poly(3-hexylthiophene): Improvement of regioregularity and energy band gap. RSC Adv..

[15] Wang W., Zhang G., Guo J., Gu Z., Hao R., Lin Z., Qian Y., Zhu M., Xia H., Peng Q. (2019). Medium-bandgap (acceptor′–donor)2acceptor-type small-molecule donors based on an asymmetric thieno[3,2-c]isochromene building block for organic solar cells with high efficiency and voltage. ACS Appl. Energy Mater..

[16] Katsumata S., Isegawa T., Okamoto T., Kubo W. (2019). Effect of metamaterial perfect absorber on device performance of PCPDTBT:PC 71 BM Solar Cell. Phys. Status Solidi (a).

[17] Kesters J., Verstappen P., Raymakers J., Vanormelingen W., Drijkoningen J., D’Haen J., Manca J.V., Lutsen L., Vanderzande D., Maes W. (2015). Enhanced Organic Solar Cell Stability by Polymer (PCPDTBT) Side Chain Functionalization. Chem. Mater..

[18] Liu J., Zhu X., Li J., Shen J., Tu G. (2016). Enhancing the thermal stability of the bulk-heterojunction photovoltaics based on P3HT/PCBM by incorporating diblock amphipathic P3HT–PEO at D/A interface. RSC Adv..

[19] Chen Y.-H., Huang P.-T., Lin K.-C., Huang Y.-J., Chen C.-T. (2012). Stabilization of poly(3-hexylthiophene)/PCBM morphology by hydroxyl group end-functionalized P3HT and its application to polymer solar cells. Org. Electron..

[20] Kim J.S., Lee Y., Lee J.H., Park J.H., Kim J.K., Cho K. (2010). High-efficiency organic solar cells based on end-functional-group-modified poly(3-hexylthiophene). Adv. Mater..

[21] Seibers Z.D., Collier G.S., Hopkins B.W., Boone E.S., Le T.P., Gomez E.D., Kilbey S.M. (2020). Tuning fullerene miscibility with porphyrin-terminated P3HTs in bulk heterojunction blends. Soft Matter.

[22] Lim B., Jo J., Na S.-I., Kim J., Kim S.-S., Kim D.-Y. (2010). A morphology controller for high-efficiency bulk-heterojunction polymer solar cells. J. Mater. Chem..

[23] Tanaka S., Rosli S.K.B., Takada K., Taniai N., Yoshitomi T., Ando H., Matsumoto K. (2017). Effects of bromination of poly(3-hexylthiophene) on the performance of bulk heterojunction solar cells. RSC Adv..

[24] Liu R., Liu Z. (2009). Polythiophene: Synthesis in aqueous medium and controllable morphology. Sci. Bull..

[25] Rassie C., Olowu R.A., Waryo T.T., Wilson L., Williams A., Baker P.G., Iwuoha E.I. (2011). Dendritic 7T-polythiophene electro-catalytic sensor system for the determination of polycyclic aromatic hydrocarbons. Int. J. Electrochem. Sci..

[26] Tamanai A., Beck S., Pucci A. (2013). Mid-infrared characterization of thiophene-based thin polymer films. Displays.

[27] Hotta S., Rughooputh S.D.D.V., Heeger A.J., Wudl F. (1987). Spectroscopic studies of soluble poly(3-alkylthienylenes). Macromolecules.

[28] Li S., Zhan L., Sun C., Zhu H., Zhou G., Yang W., Shi M., Li C.-Z., Hou J., Li Y. (2019). Highly efficient fullerene-free organic solar cells operate at near zero highest occupied molecular orbital offsets. J. Am. Chem. Soc..

[29] Zhang J., Liu W., Zhang M., Liu Y., Zhou G., Xu S., Zhang F., Zhu H., Liu F., Zhu X. (2019). Revealing the critical role of the HOMO alignment on maximizing current extraction and suppressing energy loss in organic solar cells. iScience.

[30] Scharber M.C., Mühlbacher D., Koppe M., Denk P., Waldauf C., Heeger A.J., Brabec C.J. (2006). Design rules for donors in bulk-heterojunction solar cells—Towards 10% energy-conversion efficiency. Adv. Mater..

[31] Gadisa A., Svensson M., Andersson M.R., Inganäs O. (2004). Correlation between oxidation potential and open-circuit voltage of composite solar cells based on blends of polythiophenes/ fullerene derivative. Appl. Phys. Lett..

[32] Gao B., Yao H., Hong L., Hou J. (2019). Efficient organic solar cells with a high open-circuit voltage of 1.34 V. Chin. J. Chem..

[33] Huang P.-T., Huang P.-F., Horng Y.-J., Yang C.-P. (2013). Photoluminescence study on charge transfer behavior of Poly(3-hexylthiophene) and PCBM blends. J. Chin. Chem. Soc..

[34] Otieno F., Mutuma B.K., Airo M., Ranganathan K., Erasmus R., Coville N., Wamwangi D. (2017). Enhancement of organic photovoltaic device performance via P3HT:PCBM solution heat treatment. Thin Solid Films.

[35] John S.V., Mayedwa N., Ikpo C., Molefe L., Ndipingwi M.M., Dywili N.R., Van Wyk J.L., Mapolie S., Baker P.G.L., Iwuoha E. (2016). Photoluminescence quenching of poly(octylfluorenylbenzothiadiazole) luminophore by n-type cobalt(II) salicylaldimine metallodendrimer. Synth. Met..

[36] Nismy N.A., Jayawardena K.D.G.I., Adikaari A.A.D.T., Silva S.R.P. (2011). Photoluminescence quenching in carbon nanotube-polymer/fullerene films: Carbon nanotubes as exciton dissociation centres in organic photovoltaics. Adv. Mater..

[37] Sun Y., Han Y., Liu J. (2013). Controlling PCBM aggregation in P3HT/PCBM film by a selective solvent vapor annealing. Chin. Sci. Bull..

[38] Abu-Zahra N., Algazzar M. (2013). Effect of crystallinity on the performance of P3HT/PC70BM/n-Dodecylthiol polymer solar cells. J. Sol. Energy Eng..

[39] Glatthaar M., Riede M., Keegan N., Sylvester-Hvid K., Zimmermann B., Niggemann M., Hinsch A., Gombert A. (2007). Efficiency limiting factors of organic bulk heterojunction solar cells identified by electrical impedance spectroscopy. Sol. Energy Mater. Sol. Cells.

[40] Norrman K., Krebs F.C. (2006). Lifetimes of organic photovoltaics: Using TOF-SIMS and 18O2 isotopic labelling to characterise chemical degradation mechanisms. Sol. Energy Mater. Sol. Cells.

[41] Reese M.O., Nardes A.M., Rupert B.L., Larsen R.E., Olson D.C., Lloyd M.T., Shaheen S.E., Ginley D.S., Rumbles G., Kopidakis N. (2010). Photoinduced Degradation of polymer and polymer-fullerene active layers: Experiment and theory. Adv. Funct. Mater..

[42] Seemann A., Sauermann T., Lungenschmied C., Armbruster O., Bauer S., Egelhaaf H.-J., Hauch J. (2011). Reversible and irreversible degradation of organic solar cell performance by oxygen. Sol. Energy.

[43] Cheng P., Zhan X. (2016). Stability of organic solar cells: Challenges and strategies. Chem. Soc. Rev..

[44] Campuzano S., Pedrero M., Montemayor C., Fatas E., Pingarrón J.M., Campuzano S. (2006). Characterization of alkanethiol-self-assembled monolayers-modified gold electrodes by electrochemical impedance spectroscopy. J. Electroanal. Chem..

[45] Ma X., Zhong J., Li M., Chen J., Zhang Y., Wu S., Gao X., Lu X., Liu J.-M., Liu H. (2016). Hybrid solar cells using solution-processed TiO_2_ /Sb_2_S_3_ bilayer as electron transport layer. Sol. Energy.

[46] Xu L., Lee Y.-J., Hsu J.W.P. (2014). Charge collection in bulk heterojunction organic photovoltaic devices: An impedance spectroscopy study. Appl. Phys. Lett..

[47] Zhang Y., Li L., Yuan S., Li G., Zhang W. (2013). Electrical properties of the interfaces in bulk heterojunction organic solar cells investigated by electrochemical impedance spectroscopy. Electrochim. Acta.

[48] Janek R.P., Fawcett W.R., Ulman A. (1998). Impedance Spectroscopy of Self-Assembled Monolayers on Au(111): Sodium ferrocyanide charge transfer at modified electrodes. Langmuir.

[49] Diao P., Guo M., Tong R. (2001). Characterization of defects in the formation process of self-assembled thiol monolayers by electrochemical impedance spectroscopy. J. Electroanal. Chem..

[50] Jorcin J.-B., Orazem M.E., Pébère N., Tribollet B. (2006). CPE analysis by local electrochemical impedance spectroscopy. Electrochim. Acta.

