# Supplementary material for: Synthesis and Photovoltaics of Novel 2,3,4,5-Tetrathienylthiophene-co-poly(3-hexylthiophene-2,5-diyl) Donor Polymer for Organic Solar Cell
Source: Polymers (Basel). 2020 Dec 22;13(1):2. doi: 10.3390/polym13010002 (PMC7792595; doi:10.3390/polym13010002)
Supplement: Supplementary file 1 [file polymers-13-00002-s001.pdf]

Supplementary Materials: Synthesis and Photovoltaics of Novel 2,3,4,5-Tetrathienylthiophene-co-poly(3-hexylthiophene-2,5-diyl) Donor Polymer for Organic Solar Cell

Morongwa E. Ramoroka, Siyabonga B. Mdluli, Vivian S. John-Denk, Kwena D. Modibane, Christopher J. Arendse and Emmanuel I. Iwuoha

ZnO Prepapration

ZnO solution was prepared by preparing a 5 mM zinc acetate dihydrate (≥98%, Merck (Pty) Ltd) in 2-methoxyethanol (99.8%, Merck (Pty) Ltd) with a 1:1 molar ratio of zinc acetate dihydrate to ethanolamine (≥99.0%, Merck (Pty) Ltd). The solution was stirred at 60 °C for 2 hrs. The solution was filtered with 0.45 μm filter before use [1–2].

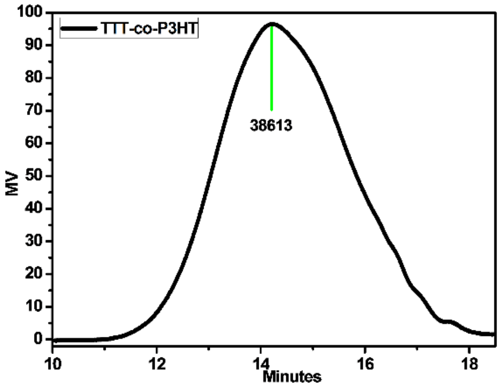

Figure. S1. Size exclusion chromatography analysis of TTT-co-P3HT in THF.

References

[1] Gómez-Núnez, A.; Alonso-Gil, S.; López, C.; Roura, P.; Vilà, A. Role of Ethanolamine on the Stability of a Sol-Gel ZnO Ink. *J. Phys. Chem. C* **2017**, *121*, 23839–23846. https://doi.org/10.1021/acs.jpcc.7b09935

[2] Kim, H.; Kwon, Y.; Choe, Y. Fabrication of nanostructured ZnO film as a hole-conducting layer of organic photovoltaic cell. *Nanoscale Res. Lett.* **2013**, *8*, 1–6. https://doi.org/10.1186/1556-276X-8-240
